# Comparing Human and AI Therapists in Behavioral Activation for Depression: Cross-Sectional Questionnaire Study

**DOI:** 10.2196/78138

**Published:** 2025-12-04

**Authors:** Inka Napiwotzki, Julian Laue, Flora Caldarone, Maximilian Idahl, Uwe Hadler, Haithem Amrani, Elisabeth Hildt, Kai G Kahl, Wolfgang Nejdl

**Affiliations:** 1 Clinic for Psychiatry, Social Psychiatry and Psychotherapy, Department of Medizinische Hochschule Hannover, Carl-Neuberg-Str 1, Hannover, 30625, Germany, 49 511 532 2495; 2 L3S Research Center, Leibniz University Hannover, Hannover, Germany; 3 Department of Humanities, Arts, and Social Sciences, Illinois Institute of Technology, Chicago, IL, United States

**Keywords:** large language model, mental health, behavioral activation, empathy, digital therapeutics, artificial intelligence, AI

## Abstract

**Background:**

Large language models (LLMs) have rapidly advanced across numerous fields, including mental health care. A shortage of trained therapists and mental health care providers has driven informal use of LLMs for therapeutic support. However, their clinical utility remains poorly defined.

**Objective:**

This study aimed to systematically evaluate and compare the therapeutic knowledge and single-turn response capabilities of LLMs versus psychotherapists in training in the context of behavioral activation (BA) therapy for depression, and to assess how both groups’ performance changed when provided with structured therapeutic training materials.

**Methods:**

Six LLMs and 8 human participants completed a questionnaire on depression and BA with 20 multiple-choice items and 10 therapy scenarios, each with 3 open-ended items, that postulated empathic response, use of validation strategies, and theory of mind capabilities. Human participants completed the questionnaire before and after a 5-hour workshop and 5-week period with learning materials. The LLMs received identical training content as context during the second test. All open-ended questions were rated on 5-point scales by 2 experts.

**Results:**

At baseline, the LLMs demonstrated higher knowledge scores than human participants (61.0 vs 52.0 out of 100 points) and were rated higher in empathy (*U*=2.0; *P*=.005; *r*=0.917), validation quality (*U*=2.5; *P*=.006; *r*=0.896), anticipation of cognition (*U*=0.0; *P*=.002; *r*=1.000), and anticipation of emotion (*U*=0.0; *P*=.002; *r*=1.000). Following BA training, the LLMs maintained their performance advantage across multiple-choice and open-ended items.

**Conclusions:**

The results suggest that LLMs may generate high-quality therapeutic single-turn responses that integrate clinical knowledge with empathetic communication. The findings hint at LLMs’ potential as valuable tools in mental health care, although further clinical trials are needed to evaluate their performance in ongoing therapeutic relationships and clinical outcomes.

## Introduction

Health care systems worldwide face a critical challenge in addressing a growing mental health crisis: while evidence-based treatments like cognitive behavioral therapy (CBT) exist, there is an acute shortage of trained professionals to deliver them. This gap affects 1 in 8 people globally who live with mental disorders, with numbers rising since the COVID-19 pandemic. Mental disorders have devastating consequences. Beyond reduced work productivity, affected individuals experience reduced social participation, physical health complications, and premature mortality [1-4].

Recent research has demonstrated the potential of large language models (LLMs) in mental health care applications [5,6]. While LLMs offer more sophisticated and natural language understanding capabilities than earlier rule-based systems, their practical implementation in therapeutic contexts remains largely unexplored [7]. However, informal therapeutic use of LLMs is already occurring. A recent study of the Replika chatbot (Luka, Inc) found users engaging in therapeutic conversations, with some reporting crisis prevention benefits [8]. These findings align with informal user discussions across social media platforms, where individuals frequently describe using general-purpose LLM platforms like ChatGPT (OpenAI) for emotional support and mental health conversations, despite these models not being designed or validated for therapeutic use (Mirzae, T, unpublished data, October 2025). This spontaneous adoption, combined with LLMs’ known risks and susceptibility to errors, underscores the critical and urgent need for rigorous evaluation to ensure their safe and effective application in therapeutic dialogue [9,10].

Researchers have explored various approaches to enhance LLMs’ therapeutic capabilities—from fine-tuning models on therapy-specific datasets to applying few-shot learning with therapist-client examples and adapting self-critique techniques [11-13]. However, these studies predominantly relied on automated evaluation methods, often using one LLM to evaluate another [12,14]. This methodological limitation points to the need for comprehensive human expert assessment.

**Figure 1.**
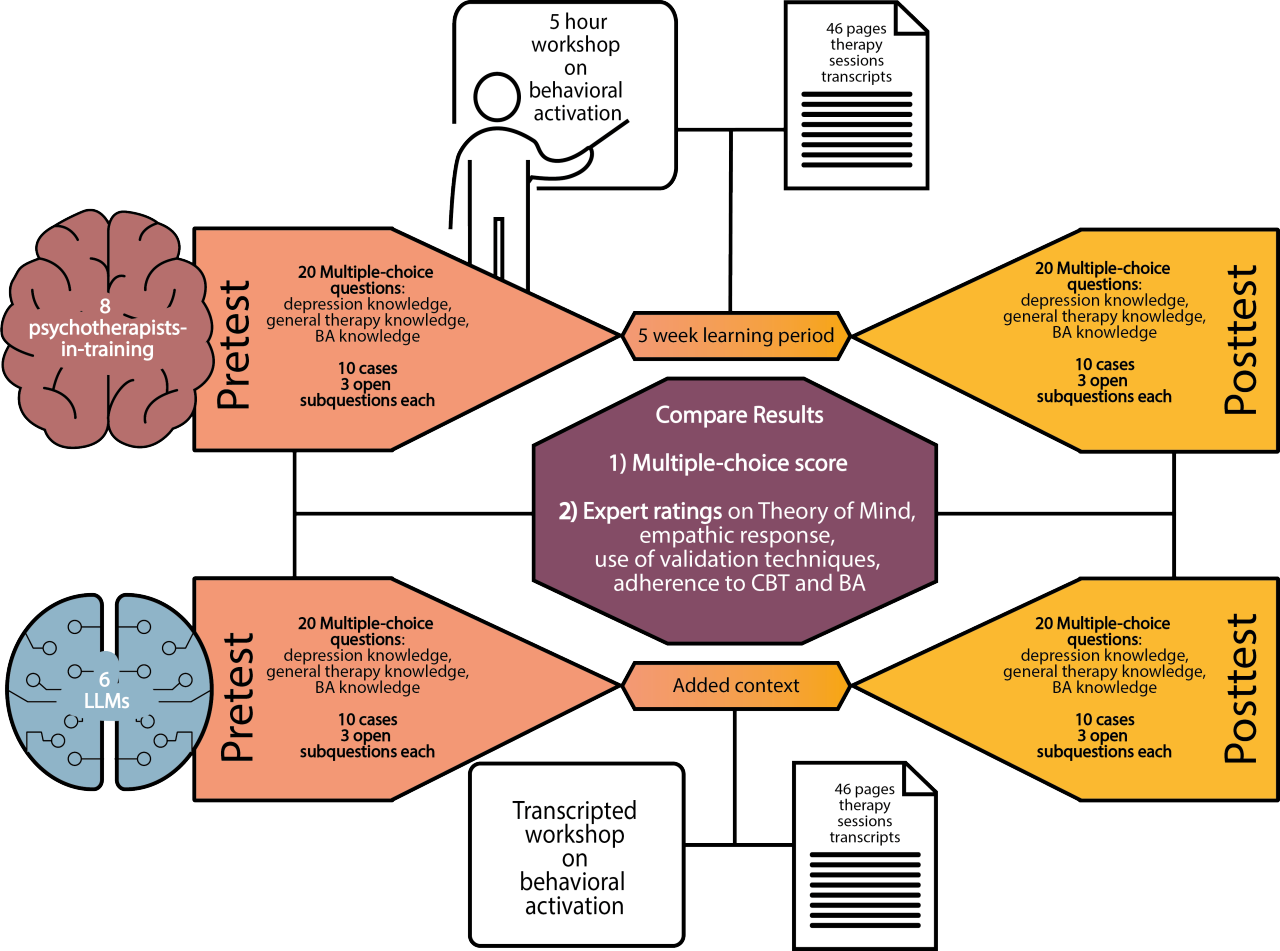
Experimental design for comparing psychotherapists in training with large language models (LLMs). Study methodology comparing psychotherapists in training (n=8, top) and LLMs (n=6, bottom) on therapeutic capabilities. Both groups completed identical assessments with multiple-choice knowledge questions and open-ended therapeutic scenarios. Human participants underwent a 5-week learning period including a BA workshop, while LLMs received equivalent information as added context during the posttest assessment. BA: behavioral activation; CBT: cognitive behavioral therapy.

To address these limitations, we present a systematic evaluation comparing 6 LLMs with 8 psychotherapists in training. Our assessment consists of 2 components. Multiple-choice questions test knowledge on depression, therapy principles, and BA, an effective therapeutic method within CBT for treating depression [15,16]. We focused on depression as it is one of the most common mental disorders [1]. Through open-ended questions, we evaluated responses to client statements, assessing empathy, use of validation strategies, and the ability to anticipate a client’s emotions and cognition. We evaluated how single-turn performance changes when LLMs are provided with therapeutic background information about BA principles and techniques, comparing this to the improvement observed in therapists after formal BA training. Figure 1 provides a visual overview of our approach. This parallel assessment reveals whether additional context enhances LLM capabilities and quantifies learning effects in human therapists.

In summary, our contributions are threefold: (1) a comprehensive evaluation questionnaire combining 20 multiple-choice and 10 open-ended questions that will be publicly available to foster further research in this domain; (2) an evaluation of LLMs in their default, publicly accessible form—the way most users currently interact with these systems; and (3) the first direct comparison (to the best of our knowledge) between LLMs and human psychotherapists in training in both therapeutic knowledge and response capabilities.

## Methods

### Participants

Participants were recruited through the mailing list of the Clinic for Psychiatry, Social Psychiatry, and Psychotherapy, as well as the Educational Institute for Psychotherapy. In total, the mailing list consisted of 102 psychotherapists in training; 10 people answered, of whom 2 dropped out of participation because they could not participate on the day of the workshop. Thus, the human sample consisted of 8 psychotherapists in training from Hannover Medical School. Six participants were in their second year of training, 1 in the first year, and 1 in the third year. At the time of this study, 7 participants were actively conducting therapy sessions with outpatients. The sample included 7 female and 1 male participant, with age ranging from 25 to 32 (mean 27.88, SD 2.2) years. At the beginning of the questionnaire, participants were asked to rate their prior knowledge of BA on a scale of 1 to 5. They rated themselves a mean 3 (SD 1.87). The LLM sample consisted of 6 different LLMs. We investigated the most well-known LLMs at the time of conducting the study, namely GPT-4 (OpenAI), GPT-4o (OpenAI), Gemini Pro 1.5 (Google), Claude Opus (Anthropic), Llama-3 70B Instruct (Meta), and Command R+ (Cohere) [17-21]. All models were accessed via OpenRouter[22] to enable a unified API interface.

### Ethical Considerations

This study was reviewed and approved by the Research Ethics Committee at the Medical University Hanover (OE 9515; approved on December 18, 2023). All participants were adults (≥18 years). Written informed consent was obtained from all participants after the aims, procedures, risks, and benefits were explained. Participants could withdraw at any time without penalty. We safeguarded privacy and confidentiality by pseudonymization, secure servers, and restricted access. No identifying information is reported. Participants received credit points for attending the workshop and 250 EUR (US $271.30) compensation. The study adhered to the Declaration of Helsinki and applicable local and national regulations.

### Variables

In Germany, psychotherapy is conducted by professionals who are trained in scientifically established methods to diagnose, heal, or mitigate disorders with pathological significance [23]. We assessed knowledge about depressive disorders, BA, and CBT with and without contextual information to evaluate baseline knowledge and learning capacity in both human therapists and LLMs.

The client-therapist working alliance represents a critical factor in symptom reduction. Essential therapist characteristics, particularly empathy and theory of mind capabilities—the ability to anticipate and comprehend a patient’s emotional and cognitive states— substantially influence the development of a therapeutic alliance [24-27]. In exploring these capabilities, Rogers [28] conceptualized psychotherapeutic empathy as a dual process encompassing both client understanding and the effective communication of this understanding, highlighting its cognitive and emotional components.

Validation strategies are fundamental communicative and alliance-building tools for affirming client experiences, communicating empathy, and fostering therapeutic engagement [29-33]. Our evaluation framework incorporates 6 validation strategies from dialectic behavioral therapy: attentiveness (actively listening and focusing on the client’s words and emotions), intermodal communication (rephrasing within the same modality, eg, “I was very sad” → “So, you were affected emotionally”), crossmodal communication (rephrasing across different modalities), biographical reference (connecting current responses to personal history), present-moment validation (affirming the appropriateness of current feelings or actions), and radical genuineness (sharing personal reflections, eg, “I would have felt the same”) [34]. These strategies provided structured criteria for evaluating validation strategy choice in the open-ended questions, assessing empathic quality, validation effectiveness, and accuracy in estimating fictional patients’ cognitive and emotional states.

### Questionnaire

We designed a pen-and-paper test in German consisting of 20 multiple-choice questions to assess knowledge on depression, practical knowledge on psychotherapy, and specific knowledge on BA. The overall score was calculated by awarding 1 point for each correctly selected or omitted answer and −1 point for incorrect selections and omissions. The maximum attainable score was 100 points for all questions, 50 points for knowledge on depression, 10 for practical knowledge, and 40 for specific knowledge on BA.

The second section entailed 10 case scenarios of psychotherapy sessions with 3 open-ended subquestions each. In the case scenarios, a fictional patient showed a reaction to a certain situation (eg, crying after a difficult therapeutic task could not be completed). First, participants were asked which emotions and cognitions they would anticipate in the patient and provide a rationale for their conclusions. In the second subquestion, the participants assumed the role of the therapist and had to outline how they would continue with the therapy. Thirdly, they were asked to formulate an answer to the patient. Responses were rated on 5 dimensions: theory of mind, empathic response, adherence to psychotherapy principles, adherence to BA techniques, and fidelity of the chosen validation strategies. The 5 rating dimensions comprised 87 subquestions in total. With 14 participants, each blinded expert evaluated 1218 individual items (87 subquestions × 14 participants).

Translated sample items from the open-ended case scenarios are provided in (Tables S1-S4 in Multimedia Appendix 1). The complete questionnaire, including all items in both German and English translation, is available in a public repository [35].

### Procedure

Human participants completed the initial questionnaire on paper (90 min) before attending a 5-hour training session on BA. The training covered BA therapeutic rationale and methods, with participants receiving handouts containing session materials and fictional therapy transcripts. These transcripts illustrated potential client-therapist challenges and provided supplementary BA information. Participants had 5 weeks to study the materials before completing the questionnaire again. The testing period was April 26 to May 31, 2024.

For the LLM evaluation, we accessed all models through OpenRouter with temperature set to 0 to ensure reproducibility, while maintaining all other model parameters at their default values. Each interaction began with the system message “Du bist ein Experte im Bereich Psychotherapie” (“You are an expert in psychotherapy”), which remained identical for pretest and posttest assessments across all models. For multiple-choice questions, models were instructed to list only correct answer options. For the case scenarios, we used a chat-based format where each model’s previous responses were preserved as distinct messages in the conversation history, rather than concatenating them into a single prompt. This meant that for each new question, the model had access to the full conversation history including its previous responses within the same case. All LLM responses were generated between April and May 2024. To ensure standardized formatting for the expert review, we manually transferred the model outputs to a Microsoft Word document, correcting any formatting inconsistencies while preserving the original content. This process created a uniform presentation format for the experts’ blind evaluation.

The same training materials provided to human participants, including session transcripts and additional BA information, were input as context for the LLMs’ posttest evaluation. Due to Llama 3.0’s limited context window of 8k tokens, it was the only model for which only a condensed summary of the BA training materials was provided. All pretest and posttest responses from both humans and LLMs were randomly aggregated and blindly rated by 2 licensed psychotherapists with expertise in BA. The raters worked independently and were blind to each other’s ratings.

### Analysis

Adherence, empathy, and goodness of fit of anticipated emotions and cognitions were rated on a scale of 1 (= “not complied”) to 5 (= “completely complied”). The mean was calculated for each group (human vs LLM) for the pretest and the posttest over all 10 questions. The theory of mind score is the mean of goodness of fit of anticipated emotions and cognitions. Six validation strategies were predefined from dialectic behavioral therapy, and the experts identified which strategies were applied in the answers. Quality of Validation was rated on a scale of 1 (= “insufficient”) to 5 (= “optimal”) and total number of applied strategies was counted for each group (human vs LLM). To ensure interrater reliability, the 2 experts jointly developed and agreed upon definitions for each rating level before beginning the evaluation. Statistical analyses used Wilcoxon signed-rank tests for within-group comparisons and Mann-Whitney *U* tests for between-group comparisons with a Bonferroni correction for multiple testing. The rank biserial correlation was used as an effect-size measure for both tests. The intraclass correlation coefficient was calculated to assess agreement between the 2 raters. This was intentional to preserve independent judgment.

## Results

Table 1 presents multiple-choice test scores for LLMs (n=6) and human psychotherapists in training (n=8) across 3 knowledge domains: depression, general therapy, and BA knowledge. At baseline, both groups demonstrated moderate domain knowledge, with LLMs showing numerically higher mean total scores (61.0, SD 7.46 vs 52.0, SD 15.43 points, *U*=16.5; *P*=.37; *r*=0.313).

**Table 1.** Mean multiple-choice assessment scores of psychotherapists in training and large language models (LLMs).

Category (score range)	Pretest (human), mean (SD)	Posttest (human), mean (SD)	Pretest (LLM), mean (SD)	Posttest (LLM), mean (SD)
Depression (0‐50)	20 (9.43)	22 (9.64)	24.33 (1.8)^a^	21.67 (3.35)
General knowledge (0‐10)	7 (2.24)	8 (2)	8.33 (1.37)	10 (0)^a^
Behavioral activation (0‐40)	25 (6.24)	24.75 (7.93)	28.33 (6.47)	32.67 (5.62)^a^
Total (0‐100)	52 (15.43)	54.75 (16.31)	61 (7.46)	64.33 (8.9)^a^

a Values indicate highest scores in each category. There were no statistically significant differences between groups or between pretest and posttest scores at *P*<.00625.

Following BA training, both groups demonstrated improvements under their respective testing conditions. Human participants, tested after a 5-week study period, showed a modest increase in mean total scores (52.0, SD 15.43 vs 54.75, SD 16.31 points; *W*=16.0; *P*=.84; *r*=0.111) with gains in depression knowledge (20.0 vs 22.0 points; *W*=9.0; *P*=.40; *r*=0.500). LLMs, evaluated with training materials as additional context, similarly showed enhanced performance with total scores improving (61.0, SD 7.46 vs 64.33, SD 8.9 points; *W*=5.0; *P*=.49; *r*=0.524), attained maximum scores in general therapy knowledge (8.3, SD 1.37 vs 10.0, SD 0.0 points; *W*=0; *P*=.06; *r*=1.000), and showed improvement in BA knowledge (28.33, SD 6.47 vs 32.67, SD 5.62 points; *W*=1.0; *P*=.08; *r*=0.905), though their depression knowledge showed a slight decrease (24.33, SD 1.8 vs 21.67, SD 3.35 points; *W*=4.0; *P*=.34; *r*=−0.619). Notably, while LLMs improved markedly in BA knowledge, human performance in this domain remained stable (25.0, SD 6.24 vs 24.75, SD 7.93 points: *W*=15.0; *P*=.74; *r*=−0.167). We observed no significant difference between pretest and posttest scores within either group. All statistical comparisons used a significance threshold of *P*<.00625.

**Figure 2.**
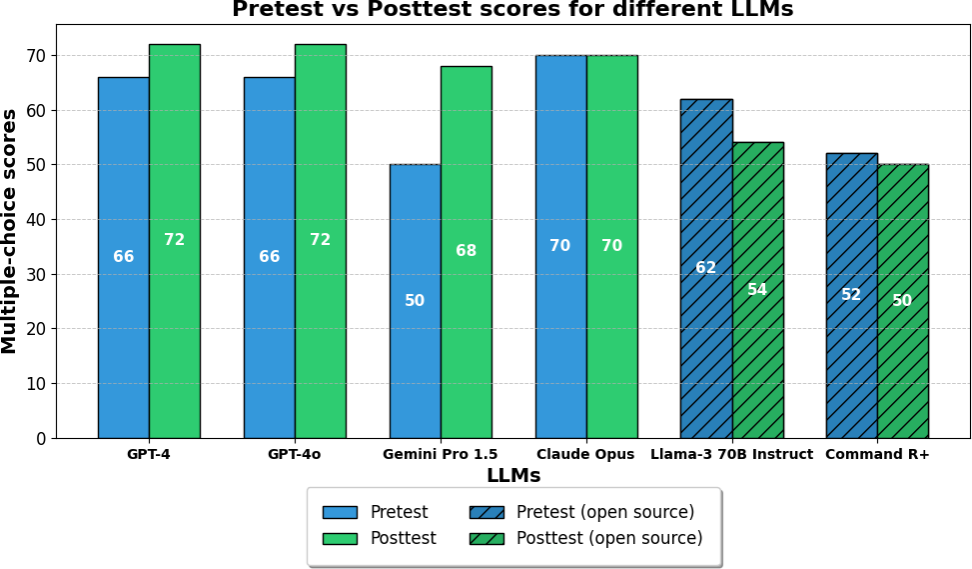
Multiple-choice assessment scores of large language models (LLMs) before and after adding behavioral activation context. Performance comparison of proprietary LLMs (solid bars) and open-source (hatched bars) on the multiple-choice knowledge assessment before (pretest) and after (posttest) receiving BA training materials as additional context.

Figure 2 shows multiple-choice pretest and posttest scores for the 6 LLMs. Proprietary models demonstrated higher performance both before and after integrating additional context, with mean scores improving from 63.0 (SD 7.68) to 70.5 (SD 1.61) points, while open-source models declined from 57.0 (SD 5) to 52.0 (SD 2) points. The limited sample size of 4 proprietary and 2 open-source models restricts formal statistical analysis, as the Mann-Whitney *U* test would yield a minimum *P*=.13, and the Wilcoxon signed-rank tests within groups would lead to a minimum 2-sided *P* value of .25 (proprietary) and .50 (open source). Both exceed conventional significance thresholds. Nonetheless, our data reveal distinct performance patterns among the proprietary models (solid bars). GPT-4 and GPT-4o improved from 66.0 to 72.0 points, Gemini Pro 1.5 showed the largest gain (50.0 vs 68.0 points), and Claude Opus remained at 70.0 points, all converging to 68 to 72 points at posttest. In contrast, both open-source models (hatched bars) showed declining scores, with Llama-3 70B Instruct falling from 62.0 to 54.0 points and Command *R*+ from 52.0 to 50.0 points. This preliminary observation indicates a performance difference, with proprietary models scoring 18.5 points higher at posttest than open-source alternatives.

In addition to the multiple-choice assessment, an open-ended questionnaire was used to evaluate more nuanced therapeutic skills. Table 2 shows the average scores for human and LLM responses. Across all pretest and posttest evaluations, LLMs achieved higher average scores than human participants on 5 psychological metrics (rated on a scale of 1 to 5). Pretest between-group comparisons showed significantly higher LLM performance for anticipation of cognition (*U*=0.0; *P*=.002; *r*=1.000) and anticipation of emotion (*U*=0.0; *P*=.002; *r*=1.000), with nonsignificant trends for adherence (*U*=4.0; *P*=.008; *r*=0.833), empathy (*U*=2.0; *P*=.005; *r*=0.917), and validation quality (*U*=2.5; *P*=.006; *r*=0.896). Both groups demonstrated improvements from pretest to posttest in adherence, empathy, and validation quality, with the largest gains in adherence (humans: 2.79, SD 0-14 vs 3.54, SD 0.43; LLMs: 3.31, SD 0.12 vs 3.96, SD 0.06). Within-group analyses revealed no significant improvements after correction, though adherence showed trends for both humans (*W*=1.0; *P*=.02; *r*=0.944) and LLMs (*W*=0.0; *P*=.03; *r*=1.000). At posttest, between-group differences were significant for adherence (*U*=2.0; *P*=.003; *r*=0.917) and empathy (*U*=1.5; *P*=.004; *r*=0.938), with nonsignificant trends for the other metrics after correction. LLMs exhibited decreases in anticipation of cognition (4.54, SD 0.30 vs 4.43, SD 0.42) and anticipation of emotion (4.69, SD 0.12 vs 4.40, SD 0.37).

**Table 2.** Mean assessment scores from the open-ended question evaluation of psychotherapists in training and large language models (LLMs).

Category	Pretest (human), mean (SD)	Posttest (human), mean (SD)	Pretest (LLM), mean (SD)	Posttest (LLM), mean (SD)	ICC^a^
Adherence	2.79 (0.14)	3.54 (0.09)	3.31 (0.12)	3.96 (0.06)^b^	0.55
Empathy	3.14 (0.43)	3.64 (0.29)	4.33 (0.09)	4.35 (0.26)^b^	0.78
Validation quality	2.78 (0.22)	3.43 (0.29)	3.78 (0.14)	4.08 (0.13)^c^	0.6
Anticipation of cognition	3.53 (0.26)	4.01 (0.26)	4.54 (0.30)^b^	4.43 (0.42)	0.67
Anticipation of emotion	3.36 (0.16)	3.80 (0.24)	4.69 (0.13)^b^	4.40 (0.37)	0.76
Average across categories	3.18 (0.10)	3.72 (0.17)	4.21 (0.07)	4.28 (0.14)^c^	0.67

a ICC: intraclass correlation coefficient.

b Indicate significant differences between human and LLM scores at the same testing point (*P*<.005).

c Values represent the highest score in each category across all conditions.

Validation strategy use showed broadly comparable patterns between LLMs and humans, with both groups reducing overall use by approximately 5%. Statistical analyses used Wilcoxon signed-rank tests for within-group comparisons and Mann-Whitney *U* tests for between-group comparisons, with Bonferroni correction applied (significance threshold *P*<.004). V1 (attentiveness) remained stable across both groups with no significant within-group changes. Both groups demonstrated similar patterns for V2 (intermodal), V3 (crossmodal), and V5 (present). V3 showed the largest increases in both groups, rising 133% for humans and 160% for LLMs, though within-group analyses revealed only trends toward significance (humans: *W*=3.0; *P*=.03; *r*=0.833; LLMs: *W*=3.0; *P*=.11; *r*=0.714). V5 decreased substantially in both groups, dropping 57% for humans and 67% for LLMs (humans: *W*=5.0; *P*=.07; *r*=-0.722; LLMs: *W*=1.5; *P*=.09; *r*=−0.857). The clearest divergence appeared in V6 (radical genuineness), where humans increased use by 38% while LLMs decreased by 14% (humans: *W*=12.0; *P*=.39; *r*=0.333; LLMs: *W*=7.5; *P*=.52; *r*=−0.286). Between-group comparisons at T1 (postintervention) showed a trend toward difference for V4 (biography; *U*=7.0; *P*=.01; *r*=0.708), although this did not survive Bonferroni correction. After correction, no comparisons reached statistical significance, indicating these patterns should be interpreted as trends rather than definitive effects (Table 3).

**Table 3.** Number of applied validation strategies of psychotherapists in training and large language models (LLMs).

Validation strategy	Pretest (human), n	Posttest (human), n	Change in human responses (%)	Pretest (LLM), n	Posttest (LLM), n	Change in LLM responses (%)
V1 (attentiveness)	39	39	0	30	30	0
V2 (intermodal communication)	11	6	−45	11	8	−27
V3 (crossmodal communication)	6	14	+133	5	13	+160
V4 (reference to client biography)	10	8	−20	10	11	+10
V5 (reference to the present)	14	6	−57	12	4	−67
V6 (radical genuineness)	8	11	+38	14	12	−14
Sum over all strategies	88	84	−5	82	78	−5

## Discussion

### Principal Findings

This study set out to evaluate whether LLMs could demonstrate therapeutic competencies comparable to psychotherapists in training, particularly in the domains of knowledge acquisition, therapeutic alliance building, and empathic communication. Consistent with recent studies showing that users perceive LLMs like Replika and ChatGPT as emotionally supportive despite not yet being designed for therapy [8,36], our findings indicated that LLMs received moderately higher ratings in certain therapeutic skills, including empathetic communication and anticipation of emotional and cognitive states. Of particular interest was the LLMs’ performance in domains traditionally associated with human capabilities: empathetic communication and emotional understanding. The observed differences between LLMs and human trainees in pretest expert-rated empathy (*U*=2.0; *P*=.005; *r*=0.917), anticipation of cognition (*U*=0.0; *P*=.002; *r*=1.000), and anticipation of emotion (*U*=0.0; *P*=.002; *r*=1.000) reveal an important distinction between producing seemingly empathetic responses and the processes behind them. Although LLMs do not experience emotions, they generated responses that trained evaluators rated as empathetic and emotionally aware, similar to human trainees. While these findings echo prior reports that LLMs can simulate empathic communication [7,12], they also raise questions of whether simulated empathy translates into effective therapeutic alliances with real patients, indicating the necessity for future research on how patients experience and respond to therapeutic interactions with LLM systems compared with human therapists.

The convergence of proprietary models’ performance on the multiple-choice assessment after exposure to training materials, reaching scores between 68 to 72 points, aligns with research findings suggesting that LLMs can rapidly integrate structured therapeutic information through few-shot prompting or fine-tuning [11-13] and may indicate similar underlying approaches to processing and integrating therapeutic knowledge across different proprietary LLMs. In contrast, the declining performance of open-source models points to potential limitations in their ability to effectively integrate and apply additional context while maintaining consistent performance for the investigated models.

These differences between proprietary and open-source models have important implications for potential therapeutic applications. The stability and convergence of proprietary models suggest they may offer more reliable performance in therapeutic contexts. However, their proprietary nature raises questions about implementation costs in real-world mental health applications, as health care providers would need to rely on more costly external APIs. The performance gap between proprietary and open-source models might present a trade-off between reliability and cost that requires careful consideration in the development of LLM-assisted mental health interventions.

Our analysis of validation strategies revealed important insights into their use patterns in both LLMs and human trainees. Across both human and LLM responses, the fundamental strategy of attentiveness remained consistent, aligning with its essential role in therapeutic alliance formation as established in previous research [24-33]. Cross-modal and intermodal communication strategies were rarely used simultaneously, likely due to the constrained nature of single-response evaluations. Subsequent training led to an increase in cross-modal communication, suggesting successful integration when explicitly emphasized in training materials. However, the observed decrease in present-moment references may indicate a shift toward more structured therapeutic interventions, as participants potentially prioritized demonstrating newly acquired technical skills over moment-to-moment emotional attunement.

### Limitations

While our findings demonstrate strong performance from LLMs across multiple therapeutic competencies, these results should be interpreted within several important constraints. First, our evaluation was limited to single-turn therapeutic responses, affecting ecological validity, since real therapeutic relationships require maintaining consistent dialogue across sessions, building upon accumulated client information, and adapting therapeutic approaches as client needs evolve. Future research needs to examine their performance in multi-turn therapeutic dialogues, their ability to maintain consistent client understanding across sessions, and adaptation to changing therapeutic needs. Additionally, the sample of 8 psychotherapists in training is too small to draw reliable inferences regarding the broader population of psychotherapists in training. The testing material should be administered to larger samples of psychotherapists and psychotherapists in training. More expert ratings might also enhance variability and thus offer more scope for interpretation.

The questionnaire was conducted in a paper-and-pencil format, which might account for shorter answers in the human sample, thus affecting the rating. The rapid evolution of AI models also affects the longevity of our findings. Even during this study’s preparation, more sophisticated models like Anthropic’s Claude 4.0 Sonnet and OpenAI’s GPT-5 were released, highlighting how quickly such comparative analyses can become outdated. The development of standardized evaluation frameworks that can account for the rapid evolution of LLM capabilities while maintaining rigorous assessment standards will be crucial for ongoing comparative analyses. Such frameworks should particularly focus on longitudinal therapeutic interactions and consistent application of therapeutic principles over time.

Another key methodological limitation affects our ability to make direct comparisons. The LLMs had access to all training materials during testing, while human participants had to rely on retained knowledge from their training period. This created an asymmetric testing environment similar to comparing open-book and closed-book exam performance. An open-book examination should be conducted and might also reflect a more realistic scenario for psychotherapists in training, since these are closer to a real-life therapeutic session where therapists have access to literature, data, and the possibility to look up information if they need to.

### Conclusions

This study provides evidence that LLMs can generate high-quality, single-turn therapeutic responses that effectively combine clinical knowledge with empathetic communication. Our findings reveal that LLMs often matched psychotherapists in training in knowledge assessments and practical therapeutic alliance-building skills. While LLMs cannot experience genuine empathy, their ability to produce responses rated as empathetic by licensed psychotherapists highlights the distinction between internal emotional experience and effective therapeutic communication.

Our findings suggest clinical applications for LLMs as supportive tools in mental health care, potentially addressing the critical shortage of trained professionals and demand in low-resource settings. They could supplement care or expand access to mental health care. However, such applications must be guided by rigorous safeguards, as the distinction between simulated and genuine human empathy has direct implications for therapeutic authenticity and patient outcomes.

Differences between proprietary and open-source LLMs emphasize issues of accessibility, equity, and sustainability. Though our results carefully suggest that proprietary models offer more reliable performance, their closed nature and cost structures risk exacerbating inequalities in access to high-quality digital mental health tools. Addressing these trade-offs will be critical in planning AI integration.

As mental health care faces growing demand, LLMs may serve as valuable supplements to human expertise, though their implementation requires careful consideration of ethical implications, therapeutic authenticity, and clinical outcomes.

## Supplementary material

10.2196/78138 Multimedia Appendix 1 Responses of Claude Opus to open-ended questions of case scenarios 2, 6, 7, and 10.

## Abbreviations

BA: behavioral activation
CBT: cognitive behavioral therapy
LLM: large language model
