# Supplementary material for: Comparing Human and AI Therapists in Behavioral Activation for Depression: Cross-Sectional Questionnaire Study
Source: JMIR Form Res. 2025 Dec 4;9:e78138. doi: 10.2196/78138 (PMC12677730; doi:10.2196/78138)
Supplement: Multimedia Appendix 1 [file formative-v9-e78138-s001.docx]

Multimedia Appendix 1

**Table S1. Pre-Test and Post-Test Claude Opus Responses to Case Scenario Question 2: Therapeutic Reasoning, Planning, and Response Formulation in Behavioral Activation Therapy (English Translation)**

| Pre-Test | Post-Test |
| --- | --- |
| a.  Cognitions: Ms. M. may have thoughts like "This is all pointless" or "I just can't feel joy anymore."  Reason: Since no pleasant feelings have arisen despite the value-oriented activity, Ms. M. may have activated negative automatic thoughts that question the effectiveness of the treatment. Such cognitions are typical of depressed patients and can lead to discouragement.    Emotions: Ms. M. is likely experiencing feelings of disappointment, frustration, and persistent low mood.  Reason: Ms. M. likely hoped that meeting her friend would improve her mood. Since this didn't happen, it triggers negative emotions. Due to her depression, she finds it difficult to experience positive feelings, which can lead to disappointment and frustration over the lack of treatment success.    b.  First, I would appreciate Ms. M. for completing the activity and validate that the lack of positive feelings can be disappointing. Then, I would explore with Ms. M. exactly what she experienced and thought during the meeting to identify potentially dysfunctional cognitions and behaviors. Together, we would consider what she can learn from this experience, for example, that behavior change is a process and that it takes several repetitions before her mood improves. We would work out what realistic expectations Ms. M. might have of the activities and how she can continue to implement them despite the lack of positive feelings. Finally, we would plan the next steps and select specific activities that Ms. M. would try until the next session to continue gaining a sense of success.    c.  Ms. M., I can fully understand that you are disappointed and discouraged because the meeting with your friend did not result in the positive feelings you had hoped for. It is completely normal that you do not feel better immediately after the activity, as changing your mood through pleasant activities takes time and repeated practice. Let's find out together what can help you continue to implement the activities, even if the positive feelings don't arise immediately. | a.  Cognitions: Ms. M. may think that the activity did not have the desired effect and that she is not making progress in therapy. She may question her abilities and progress.  Reason: If an activity does not have the expected positive effect, doubts and negative thoughts may arise regarding her own progress and the effectiveness of the therapy. This is especially likely if the patient initially had high expectations of the effects.    Emotions: Ms. M. may feel disappointment, frustration, and possibly hopelessness. Shame or guilt about "failure" are also conceivable.  Reason: The lack of the hoped-for positive feelings after the activity can lead to disappointment and frustration. If the patient feels that the therapy is not working, this can trigger hopelessness. Shame and guilt may arise if she believes she is to blame for the lack of success.    b. First, I would like to appreciate Ms. M. for her openness and for completing the homework. Then I would validate her disappointment and normalize that value-oriented activities don't always have to feel immediately positive. Together, we would analyze the situation more closely to determine what might have influenced her experience of the activity (e.g., expectations, context, duration). I would then work with Ms. M. to determine what conclusions she can draw from it and how she wants to proceed. I would emphasize that this is a process and that we will continue with it together.    c. Ms. M., I fully understand that you are disappointed and frustrated because the meeting with your friend didn't have the desired effect. It's perfectly normal to have imagined things differently and to now doubt yourself and your progress. Let's take a closer look together at what might have influenced the situation and consider what conclusions we can draw from it. |

**Table S2. Pre-Test and Post-Test Claude Opus Responses to Case Scenario Question 6: Therapeutic Reasoning, Planning, and Response Formulation in Behavioral Activation Therapy (English Translation)**

| Pre-Test | Post-Test |
| --- | --- |
| a.  Cognitions: I would most likely not expect Mr. O. to have positive, optimistic thoughts about himself, his performance, and his future while at work.  Reason: Depression is often accompanied by a negative view of himself, his environment, and his future. Despite his activity at work, it is likely that Mr. O. is accompanied by dysfunctional, pessimistic thoughts and perceives few senses of achievement.    Emotions: I would rather not expect Mr. O. to experience joy, pride, and satisfaction with his work.  Reason: Due to his depressive symptoms, it is likely that Mr. O.'s positive emotions are significantly reduced. Instead, feelings of sadness, hopelessness, and emotional numbness are to be expected, even if he has a regular job.    b.  I would first like to thank Mr. O. for his openness and for addressing his concerns. I would then explain that Behavioral Activation is not just about increasing activity, but primarily about finding activities that bring joy, enhance a sense of accomplishment, and evoke positive emotions. Together, we would analyze the activity log more closely to determine if there are other potentially positively reinforcing activities besides work. If not, we would consider what activities Mr. O. used to enjoy or what new activities he could try. I would emphasize the importance of making realistic and concrete plans and not overextending yourself.    c.  Mr. O., I can well understand that you are wondering how Behavioral Activation can help you if you are not inactive. Let's take a closer look at your activity log together and determine if there are other activities besides work that you used to enjoy or that you would like to try. Behavioral Activation is not just about becoming more active, but primarily about finding activities that can improve your well-being and mood. | a.  Cognitions: I would most likely not anticipate positive, motivating thoughts like "I enjoy my work and it gives me energy" from Mr. O. while at work.  Reason: Since Mr. O. sleeps most of the time after work, work seems to exhaust him and bring him little joy. The work does not seem value-oriented or reinforcing.    Emotions: I would most likely not anticipate positive, pleasant emotions like joy, satisfaction, or pride from Mr. O. while at work.  Reason: Mr. O.'s behavior of primarily sleeping after work suggests that work triggers negative emotions such as overwhelm, frustration, or tension in him and is not experienced as rewarding.    b.  I would first thank Mr. O. for his question and assure him that this is a very important question. I would then explain to him that activity is not synonymous with value-oriented, positively reinforcing activity, and that behavioral activation is about identifying and developing precisely such activities. I would suggest that we look at his values ​​together to determine which activities are truly important to him and would benefit him in the long term. I would emphasize that while work is an important area of ​​life, it is not the only one, and that there are various ways he can get closer to his values. Finally, I would reassure him that we will proceed step by step and that he can ask questions at any time.    c.  Mr. O., thank you for your important question. Behavioral activation is about finding activities that are personally important to you and that will have a positive long-term impact on your well-being. Let's explore together which activities these might be for you and how we can integrate them step by step into your everyday life. |

**Table S3. Pre-Test and Post-Test Claude Opus Responses to Case Scenario Question 7: Therapeutic Reasoning, Planning, and Response Formulation in Behavioral Activation Therapy (English Translation)**

| Pre-Test | Post-Test |
| --- | --- |
| a.  Cognitions: Ms. M. might think, "It's silly to plan something like that. I don't usually do that. The therapist doesn't understand me."  Reason: Ms. M. isn't used to consciously planning activities and finds it unnatural. She might question the usefulness of the intervention and feel misunderstood by the therapist.    Emotions: Ms. M. might feel discomfort, mild frustration, and insecurity.  Reason: Planning activities is an unfamiliar approach for Ms. M., taking her out of her comfort zone. This can lead to discomfort and insecurity. She might also feel frustrated because she doesn't (yet) understand the purpose of the intervention and may not feel sufficiently understood.    b.  I would first thank Ms. M. for her open feedback and show understanding for her feelings. I would then again explain the importance and purpose of activity planning within the framework of Behavioral Activation and emphasize that, while it can often seem strange and artificial at first, it is an important step in the therapy process. I would encourage Ms. M. to try it anyway and consider together how we can make the planning more pleasant and natural for her, for example, by using less detailed planning or incorporating activities she previously enjoyed. I would emphasize that we can adjust the planning at any time if it doesn't feel right and that we will evaluate how she copes with it together in the next sessions.    c.  Ms. M., I can well understand that planning activities feels strange and artificial to you, as you haven't done it in this way before. Let's consider together how we can make the planning more coherent and adapt it to your needs. It is important that you feel comfortable with it and that we proceed in small steps to give you the opportunity to get used to this new approach. | a.  Cognitions: Ms. M. probably thinks that planning activities isn't natural and doubts whether it will help her. She compares the planning with her previous approach and realizes that she has never done anything like this before.  Reason: Ms. M. isn't used to planning activities in detail. Due to her depression, she finds it difficult to imagine that planning could help her, as she has managed without it so far.    Emotions: Ms. M. probably feels uncertainty, doubt, and possibly even frustration.  Reason: Detailed planning is new to Ms. M., so it's understandable that she feels unsure whether she can implement it. The doubts arise from the fact that she can't assess whether planning will actually help her. Frustration may arise because Ms. M. may feel that planning represents an additional burden and doesn't (yet) understand the purpose behind it.    b.  I would first thank Ms. M. for her openness and let her know that I understand her concerns. Then, I would explain to her that detailed planning is an important component of therapy to provide structure and orientation and increase the likelihood that she will actually implement the planned activities. I would emphasize that it's normal for planning to feel unfamiliar at first and encourage her to try it anyway. Together, we could consider what might help her feel more comfortable with planning, for example, by approaching the planning step by step or by letting her decide which activities she wants to plan first. Finally, I would reassure her that we will continue to address her planning experiences in future sessions and make adjustments as needed.    c.  Ms. M., I can very well understand that the detailed planning of activities feels unfamiliar and artificial to you; many people feel this way. At the same time, planning is an important component of therapy that can help you gain structure and orientation and increase the likelihood that you will actually implement the planned activities. Let's think together about what might help you feel more comfortable with planning, for example by approaching the planning step by step or by letting you decide for yourself which activities you would like to start with. |

**Table S4. Pre-Test and Post-Test Claude Opus Responses to Case Scenario Question 10: Therapeutic Reasoning, Planning, and Response Formulation in Behavioral Activation Therapy (English Translation)**

| Pre-Test | Post-Test |
| --- | --- |
| a.  Cognitions: Ms. B. might have thoughts like "I can't jog as long as I used to. I'm not as efficient anymore." or "I'll never be as fit as I was before the depression."  Reason: Ms. B. compares her current performance with her performance before the depressive phase. This comparison is negative, which can lead to self-deprecating thoughts and doubts about her own abilities.    Emotions: Ms. B. might experience feelings of frustration, disappointment, and sadness.  Reason: The negative thoughts and the comparison with the time before the depression can lead Ms. B. to a feeling of failure and dissatisfaction with herself. This can trigger frustration and sadness because she hasn't been able to meet her own expectations.    b.  In the session, I would first appreciate Ms. B. for her courage and willingness to try jogging. Then I would invite her to explore the situation in more detail: What thoughts and feelings arose during and after the jog? Together, we would challenge these thoughts and look for more helpful alternatives, e.g., "I've taken a first step and can be proud of it, even if I haven't reached my goal yet." We would consider how Ms. B. can set her goals more realistically and be more compassionate towards herself. Finally, we would plan how she would like to structure her jogging activity in the coming week, e.g., starting with shorter intervals and then increasing.    c.  Ms. B., first of all, I would like to say that I think it's very brave of you to try jogging, even if it didn't work out quite as you had imagined. Let's explore together what thoughts and feelings arose in the situation and how we can deal with them. I'm sure we can find a way for you to approach your goals at a pace that works for you. | a.  Cognitions: Ms. B. may have thoughts like "I can't jog as long as I used to. I'm no longer efficient." or "Everything will never be the same again."  Reason: Comparing herself to the time before the depressive episode and realizing that she currently has less stamina for jogging than before could trigger dysfunctional cognitions. These could relate to herself (e.g., "I'm a failure.") or the future (e.g., "It will never get better.").    Emotions: Ms. B. may experience feelings such as frustration, sadness, disappointment, and hopelessness.  Reason: The dysfunctional cognitions and the perceived failure of comparing her current jogging performance with her previous performance could trigger negative emotions. In particular, frustration and disappointment about her own performance, but also sadness and hopelessness regarding the future, are conceivable.    b.  First, I would appreciate Ms. B.'s courage and effort in trying jogging. Then, I would ask her about her specific thoughts and feelings during and after jogging to explore her cognitions and emotions. I would then psychoeducate Ms. B. to explain that it's completely normal not to be able to perform at the same level as before after a prolonged depressive phase, and that it's important not to compare yourself to the time before the depressive phase, but rather to focus on the current situation. Together, we would work out that even the 15-minute jog was a success and consider how to structure her jogging in the future so that Ms. B. doesn't overtax herself (e.g., adjust the duration and frequency). Finally, I would discuss with Ms. B. that she can be proud of herself for having taken this first step and that we will work in small steps to help her slowly increase her endurance again.    c.  Ms. B., first of all, I want to say that I think it's very impressive that you pulled yourself together and went jogging. That requires a lot of effort in your current situation. Even if you were able to jog for longer periods before, it's perfectly normal not to be able to perform at the same level straight away after a prolonged bout of depression, and the 15 minutes you managed are an important first success. Let's think together about how we can structure your jogging in the near future so that it doesn't feel so overwhelming and frustrating, and you can slowly regain your previous stamina. |
